# In the wild hybridization of annual *Datura* species as unveiled by morphological and molecular comparisons

**DOI:** 10.1186/2241-5793-21-11

**Published:** 2014-06-23

**Authors:** Ioannis T Tsialtas, Efstathia Patelou, Nikolaos S Kaloumenos, Photini V Mylona, Alexios Polidoros, Georgios Menexes, Ilias G Eleftherohorinos

**Affiliations:** Faculty of Agriculture, Laboratory of Agronomy, Aristotle University of Thessaloniki, 541 24 Thessaloniki, Greece; Faculty of Agriculture, Laboratory of Genetics and Plant Breeding, Aristotle University of Thessaloniki, 541 24 Thessaloniki, Greece; Biological Sciences, Syngenta, Jealott’s Hill International Research Centre, Bracknell, Berkshire, RG42 6EY UK; ELGO-“Demetra”, Agricultural Research Center of Northern Greece, 570 01 Thermi, Greece

**Keywords:** Fierce thornapple, Hybrids, Jimsonweed, Solanaceae, Self fertilization

## Abstract

**Background:**

The present work aimed to verify whether intermediate variants were natural crosses between *Datura* species (*D. stramonium* forms and *D. ferox*). Their existence has been long ago insinuated but has not been studied using morphological features and molecular tools. The variants differed in stem coloring, upper bearing forks, and fruit characters.

**Results:**

Principal Components Analysis of 11 morphological characteristics showed that *D. ferox* and *D. stramonium* (forms *stramonium* and *tatula*) were quite different and the putative hybrids were intermittent. The *D. ferox* × *D. stramonium* f. *tatula* was closer to the latter of its parents. Sequencing analysis revealed identical amplified trnL intron in all variants and a 100% homology with *D. stramonium* accession number EU580984.1 suggested that this plastid cannot discern *Datura* variants. However, genomic analysis with URP markers indicated that the hybrids had >60% genetic makeup similarity with both parents suggesting that the intermediate variants were putative inter-specific hybrids. Moreover, the dendrogram stemmed from cluster analysis of the fingerprint profile of variants placed *D. stramonium* and *D. ferox* in different branches indicating their genetic differentiation from each other as well as from their hybrids.

**Conclusions:**

The findings suggest that the natural hybridization of annual *Datura* species occurs. Extrapolating, this hybridization could be the first step for speciation. More possibly, it can alter population composition, its weediness and adaptability to local conditions.

## Background

Genus *Datura,* family Solanaceae, consists of nine (annual and tree) species, originating from the New and Old World [1]. In Greece, *Datura* species are considered to be invasive [2], known since antiquity for their narcotic and medicinal actions [3, 4]. Nowadays in Greece, *D. stramonium* L. forms (f. *stramonium* and f. *tatula*), *D. ferox* L., and *D. innoxia* L. coexist in mixed populations in various combinations and relative ratios. *Datura stramonium* f. *stramonium* is the most common variant found as spring weed in fields, roadsides and dumps and usually coexists with the recently identified to occur in Greece *D. stramonium* f. *tatula* L. [5]. Fierce thornapple (*D. ferox* L.) is the dominant *Datura* species in some sites in northern Greece where it shares habitat with *D. stramonium*. Finally, *D. innoxia*, commonly used as ornamental, is found as feral, usually, in dumps [5].

*Datura stramonium* is a predominantly self-fertilized species but cross-pollination is feasible to some extent by insects like hawkmoths and honeybees [6]. The predominance of self-fertilization is ascribed to anther-stigma overlapping and results in inbreeding [7, 8], which found to reduce vigor and increase herbivory damages [9]. However, within *Datura* populations, there are plants showing herkogamy (anther-stigma separation), which permits outcrossing at low rates ranging from 1.3% to 8.5% [6, 10]. The existence of anthocyanin in the purple-stemmed, violet-flowered *D. stramonium* f. *tatula* does not affect outcrossing rates [11].

In tree *Datura* species, inter-specific crossing is feasible (*D. aurea* × *D. candida*) descending hybrids with increased alkaloid content, which could be of economic interest [12]. Regarding annual species, Husaini & Iwo [13] reported the existence of cytological compatibility between *D. stramonium* and *D. ferox*. Weaver & Warwick [14], reporting the findings of Rietsema [15], stated that these two *Datura* species are the only annual species, which gave identified hybrids in the wild, collected in South America. Since then, no relative report exists, making morphological and molecular confirmation of analogous findings necessary. Given that crosses between the annual *Datura* species yielded viable seeds [15], possible outcrossing in mixed communities would diversify the existing populations to new ones, with unpredicted features regarding competitiveness, resistance to herbivores, alkaloid content and herbicide tolerance.

During September 2011, in mixed *Datura* swards in northern Greece, specimens were found showing morphological features intermediate to those of the coexisting *D. stramonium* forms and *D. ferox*. Thus, the aim of this study was to verify naturally occurring *Datura* crosses and unmask the possible hybridization by morphological features and molecular tools.

## Results

### Morphological analysis

The five variants differed significantly for all the characteristics determined (Table 1). Regarding stem color (SC), the putative *D. ferox* × f. *stramonium* hybrid had green stem resembling f. *stramonium*, while the putative *D. ferox* × f. *tatula* hybrid followed the stem coloring of f. *tatula* (Table 1).

**Table 1:** **Means (±standard errors) for the characteristics determined** ***in situ*** **in the five** ***Datura*** **variants**

Variants	Characteristics											
	Spines/capsule	CL (mm)	CW (mm)	SSL (mm)	MSL (mm)	LSL (mm)	LL (cm)	LW (cm)	LL/LW	CoL (cm)	CaL (cm)	SC
*D. stramonium* f. *stramonium*	295.17^a^ (±7.53)	33.33^b^ (±0.42)	26.00^a^ (±0.26)	0.58^c^ (±0.08)	2.50^d^ (±0.22)	6.00^d^ (±0.26)	16.72^a^ (±0.39)	12.47^a^ (±0.38)	1.35^bc^ (±0.06)	9.45^a^ (±0.12)	4.78^a^ (±0.18)	Green
*D. stramonium* f. *tatula*	212.33^b^ (±8.23)	38.67^a^ (±0.61)	28.17^a^ (±1.25)	2.33^a^ (±0.33)	4.50^c^ (±0.22)	17.50^b^ (±0.22)	13.37^b^ (±0.51)	8.85^c^ (±0.38)	1.51^a^ (±0.02)	9.60^a^ (±0.17)	4.73^a^ (±0.18)	Purple
*D. ferox*	49.00^e^ (±2.85)	37.83^a^ (±1.54)	25.67^a^ (±1.89)	2.67^a^ (±0.33)	13.67^a^ (±0.99)	24.83^a^ (±1.97)	12.23^b^ (±0.45)	11.70^a^ (±0.37)	1.05^d^ (±0.02)	6.70^b^ (±0.42)	4.28^ab^ (±0.29)	Grey-green
*D. ferox* × f. *stramonium*	142.67^d^ (±3.16)	28.67^c^ (±0.76)	18.00^b^ (±0.45)	1.50^b^ (±0.18)	6.67^b^ (±0.71)	12.17^c^ (±0.70)	12.67^b^ (±0.37)	10.22^b^ (±0.49)	1.25^c^ (±0.04)	8.98^a^ (±0.15)	3.80^b^ (±0.16)	Green
*D. ferox* × f. *tatula*	182.83^c^ (±7.23)	30.83^c^ (±0.16)	26.33^a^ (±0.42)	1.00^bc^ (±0.02)	5.50^cd^ (±0.22)	9.83^c^ (±0.47)	10.80^c^ (±0.25)	7.50^d^ (±0.40)	1.45^ab^ (±0.05)	Nv	Nv	Purple

Data from six replicates. CL: capsule length; CW: capsule width; SSL: short spike length; MSL: medium spike length; LSL: long spike length; LL: leaf length; LW: leaf width; CoL: corolla length; CaL: calyx length; SC: stem color. Within each column, means followed by common letter(s) did not differ significantly at *p* < 0.05 according to the Duncan’s multiple range test. nv: no value for this character.

The use of PCA with varimax rotation of the 11 morphological characteristics revealed two significant components which extracted the 70% of the total variance (Table 2). The component PC1 with eigenvalue 4.4 extracted the 40% of the total variance and the second component, PC2, with eigenvalue 3.3, the 30%. According to the results of parallel analysis, the critical value for significant eigenvalues was 2.1. Table 2 presents the loadings of the 11 morphological characteristics on the two components.

**Table 2:** **PCA results for the selected characteristics of 30** ***Datura*** **individuals**

	Components	
Morphological characteristics	PC1	PC2
Long spine length (LSL)	0.93	-0.02
Medium spine length (MSL)	0.90	-0.31
Spines/capsule	-0.89	0.20
Short spine length (SSL)	0.79	0.03
Purple	-0.01	0.97
Leaf width (LW)	-0.02	-0.83
Length/width (LL/LW)	-0.54	0.74
Green	-0.67	-0.68
Capsule length (CL)	0.49	0.22
Capsule width (CW)	0.09	0.55
Leaf length (LL)	-0.57	-0.41
Eigenvalue	4.4	3.3
Extracted variance	40%	30%
Kaiser-Meyer-Olkin measure	0.66	
Average communality	0.70	

Bartlett’s Test of Sphericity: *χ* ^2^(55) = 423.13, *p* < 0.001.

The PC1 was mainly correlated (positively or negatively) with long spine length, medium spine length, spines/capsule, short spine length, capsule length, and leaf length (Table 2). The PC2 was mainly loaded (positively or negatively) by the purple color, leaf width, length/width, and capsule width. The green color had almost the same negative loading on both components.

**Figure 1:**
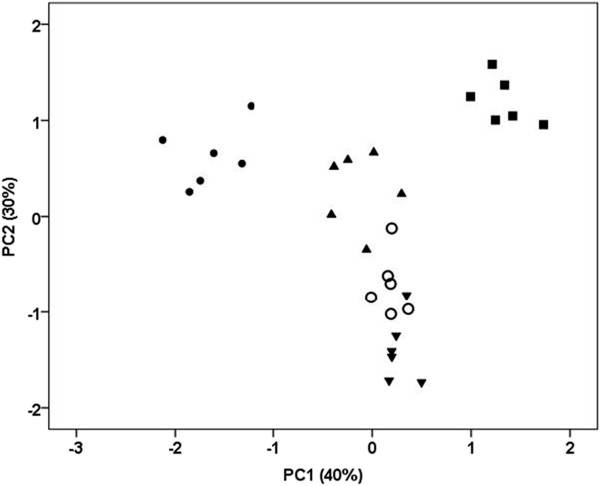
**Plot of** ***Datura*** **individuals by first and second factorial scores derived from PCA.** ● *D. ferox*, ■ f. *stramonium*, ○ f. *tatula*, ▲ *D. ferox* × f. *stramonium* and ▼ *D. ferox* × f. *tatula*.

The projection of the 30 plant individuals on the 1 × 2 factorial plane derived from PCA indicated that the first component PC1 clearly separated the *D. ferox* from f. *stramonium* individuals while *D. ferox* × f. *stramonium* plants were projected around the origin almost in the middle of their two parents (Figure 1). The second component PC2 distinguished *D. ferox* and f. *stramonium* individuals from the f. *tatula* and *D. ferox* × f. *tatula* plants. The *D. ferox* × f. *tatula* variants were most similar with one of their parents, f. *tatula*. The individuals of *D. ferox*, f. *stramonium*, and *D. ferox* × f. *stramonium* showed greater variability along the PC1 axis than the f. *tatula* and *D. ferox* × f. *tatula* plants, which had very little variability on the same axis; in contrast they showed some variability along the PC2 axis.

### Molecular analysis

**Figure 2:**
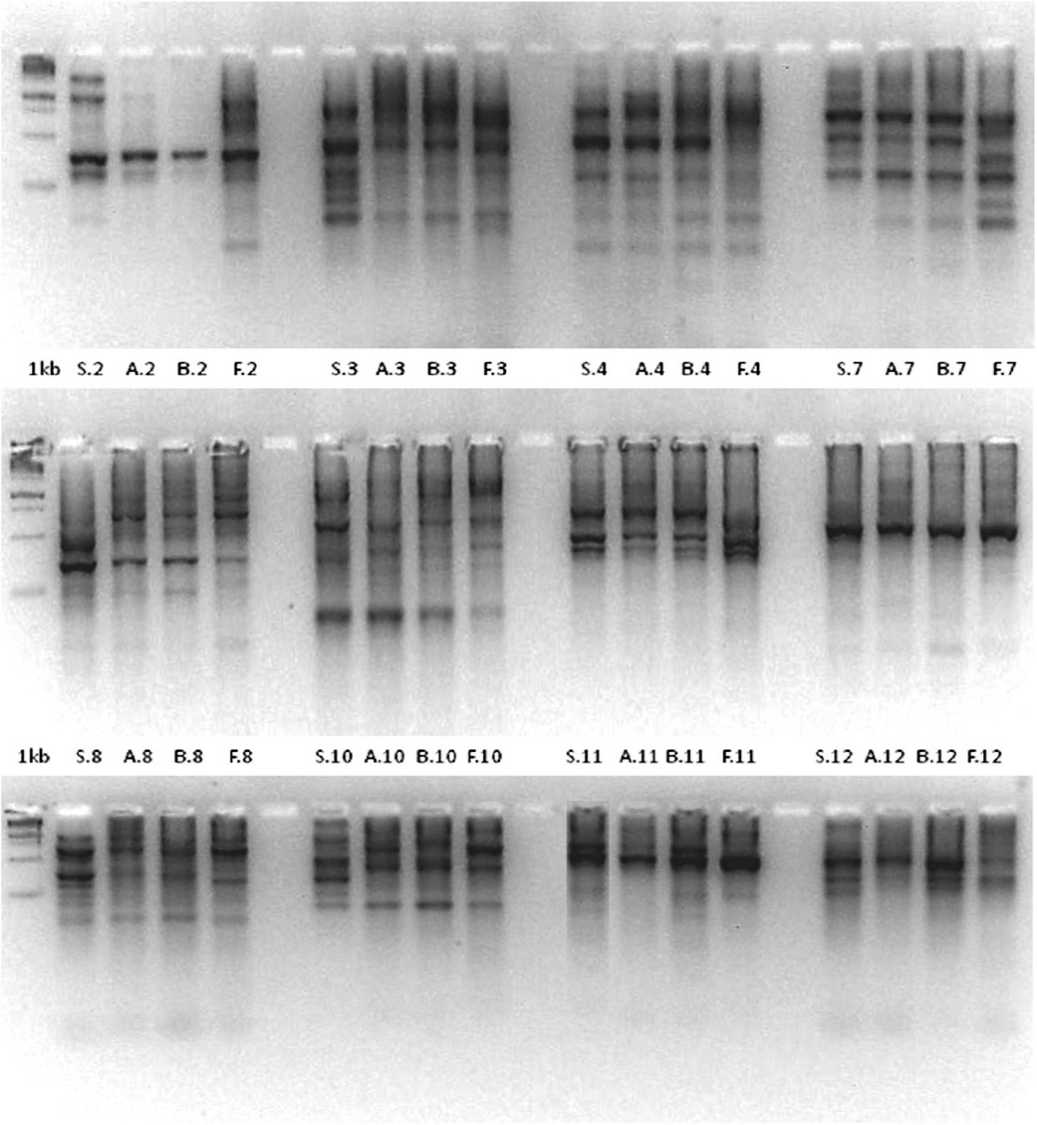
**PCR products of the** ***Datura*** **variants amplified using the URP primers.** Each lane is labeled according to the template and primer used for amplification. Letters represent variants followed by dot and a number that represents primer. Variant labels are S: *D. stramonium*; F: *D. ferox*; A: putative *D. ferox* × f. *tatula*; B: putative *D. ferox* × f. *stramonium*. URP primers used (1–12) are shown in Table 5. Molecular marker is the 1 kb DNA ladder.

The PCR fingerprinting, using all the primers and DNA, detected 63 clear and scorable bands in the samples including *D. stramonium*, *D. ferox*, and the two putative inter-specific hybrids (Figure 2). The band patterns of the *D. stramonium* forms were identical and only results from one form are shown. The URP primers produced multiple bands in all variants, varying in size from about 100 bp to more than 3000 bp. Polymorphic as well as monomorphic bands were revealed with 11 from the 12 URP primers. Only primer URP13R (number 7 in Figure 2) produced a single monomorphic band in all samples. Out of 63 scorable bands, 37 bands (58.73%) were found to be polymorphic (present in one to three variants) while 26 bands (41.27%) were monomorphic (present in all variants). Totally, 20 polymorphic bands (31.75%) were present in only one variant, while six were present in two (9.5%) and 11 in three variants (17.5%).

The 12 different primers generated various banding patterns, ranging from 1 to 9. The maximum number of scorable bands (9) was observed in primers 1F and 6R. Primer 1F also produced the highest number (8) of polymorphic bands (88.88%) and thus, it showed the higher level of polymorphism. On the other hand, the primer 13R produced a single monomorphic band. The numbers of total, polymorphic, and monomorphic bands indicated an average of 5.25 bands per primer of which an average of 3.08 were polymorphic and 2.16 monomorphic (Table 3). The URP band patterns were used to determine the genetic distances between *Datura* variants. The genetic similarity for pairs of variants was calculated using the Jaccard’s coefficient. The similarity matrix based on all possible pairs had a similarity range from 46% to 86% (Table 4). The lower similarity value of 46% indicating the higher distance was between *D. stramonium* and *D. ferox*, while the higher similarity value of 86% was between the two putative inter-specific hybrids indicating the closer relationship (smaller genetic distance).

**Table 3:** **Characteristics of the bands amplified using 12 URP primers in the examined variants**

No	Primers	No of bands	Polymorphic	Monomorphic
1	URP1F	9	8	1
2	URP2F	6	5	1
3	URP2R	4	0	4
4	URP4R	5	3	2
5	URP6R	9	6	3
6	URP9F	5	1	4
7	URP13R	1	0	1
8	URP17R	6	3	3
9	URP25F	6	3	3
10	URP30F	6	5	1
11	URP32F	3	2	1
12	URP38F	3	1	2
TOTAL		63	37	26
Average/primer		5.25	3.08	2.16

**Table 4:** **Molecular data proximity matrix constructed from the Jaccard's similarity coefficients for the variants**

Variants	Jaccard similarity coefficients			
	S	A	B	F
**S**	1.00			
**A**	0.60	1.00		
**B**	0.67	0.86	1.00	
**F**	0.46	0.62	0.62	1.00

S: *D. stramonium*; F: *D. ferox*; A: *D. ferox* × f. *tatula*; B: *D. ferox* × f. *stramonium*.

**Figure 3:**
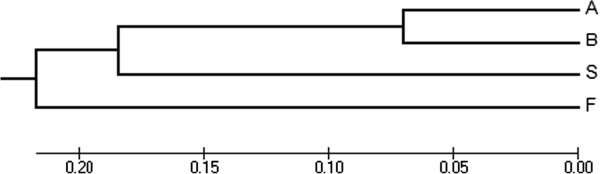
**Dendrogram showing relationships of the** ***Datura*** **variants according to their fingerprinting profile.** The tree was constructed from the Jaccard dissimilarity matrix using the UPGMA method of the MEGA5 software. S: *D. stramonium*; F: *D. ferox*; A: *D. ferox* × f. *tatula*; B: *D. ferox* × f. *stramonium*.

The dendrogram obtained from the cluster analysis of the examined fingerprint profile of the variants showed grouping of the putative hybrids in one cluster (Figure 3). The position of *D. stramonium* and *D. ferox* in different branches of the tree indicates their genetic differentiation from each other as well as from the putative hybrids.

The sequencing analysis revealed identical amplified trnL intron in all variants. BLAST similarity search of the GenBank for homologous sequences revealed that a sequence of *D. stramonium* with accession number EU580984.1 had 100% homology over the 505 nucleotide amplified region (data not shown).

## Discussion

In the wild, inter-specific hybridization is a common phenomenon especially among plant species and may become the first step in the process of speciation [16, 17]. Sympatric coexistence, intermediate characters, inter-fertility, and biochemical additivity are criteria determining the feasibility of inter-specific hybridization [18], and several of them were met in the present study.

The first clue for this work was the intermediate characters of specimens found to coexist with their putative parents (*D. ferox* and *D. stramonium*). Despite the high percentage of self-fertilization, annual *Datura* species are cytological compatible [13], show herkogamy [6, 10] and attract insect pollinators [6]. In particular, under Greek conditions, f. *tatula* is highly attractive for honeybees [5].

The possible existence of hybrids of annual *Datura* species in the wild goes back to 1950’s [15]. Nevertheless, exempting its insinuation, no proof has been provided. Nowadays, a combination of morphological and molecular characters is used to provide evidence for the existence of a putative hybrid [19–23]. Selecting an adequate number, in statistical terms (six specimens per variant), of a coherent plant cohort, we found out that the *Datura* variants were mainly different in stem coloring, upper bearing forks, and fruit characters that were determined. PCA revealed that *D. ferox* and f. *stramonium* were different and their putative hybrid was intermediate. The other putative hybrid, *D. ferox* × f. *tatula*, was closer to the latter of its putative parents. However, morphological intermediates can also be derived from convergent evolution or environmental selection and this makes ambiguous the confirmation of putative hybrid individuals based solely on morphological evidence. Use of molecular methods provides a number of advantages over morphological analysis, among others being the large number of available markers, their apparent selective neutrality, and the low levels of non-heritable variation [24]. Ideally, additive molecular markers displaying co-dominant inheritance (e.g. SSR), combined with morphological analysis, should be applied to decipher putative hybridization. The lack of such SSR markers for *Datura* species makes their development cumbersome and time-consuming. Nevertheless, genetic analyses by dominant markers, like RAPDs and AFLPs, solitary or flanked by morphological analysis, have been routinely employed with ultimate success in inter-specific hybrid verification [25–27].

In the present work, genetic distances of the examined variants were estimated using URP primers, which have been proved valuable in genomic fingerprinting of a variety of organisms including plants, animals, and microorganisms [28]. The URP markers were selected for their advantages over RAPDs (high annealing temperature leading to high specificity and reproducibility) and AFLPs (less laborious and equally specific) in genetic analysis. The applied URP primers were employed for first time in *Datura* species genetic fingerprinting, and proved to be successful in amplification of polymorphic fragments that are sufficient to discriminate their phylogenetic origin.

The fact that the sequencing analysis revealed identical amplified trnL intron in all variants and a 100% homology with *D. stramonium* accession number EU580984.1 [29] indicates that the typically used plant plastid phylogenetic marker trnL intron is not useful for discrimination, since it is identical between *D. stramonium* and *D. ferox* and, consequently, in their putative inter-specific hybrids.

The molecular characterization of the different *Datura* accessions provided the most compelling evidence on the genetic relationships and genetic makeup of the putative inter-specific hybrids. Using Jaccard’s coefficient for comparison, the similarities determined between the bands of the examined variants bolstered the possibility of putative hybrids (*D. ferox* × f. *stramonium* and *D. ferox* × f. *tatula*) to be indeed hybrids between annual *Datura* species. *Datura ferox* was equally related to both of the putative hybrids with 62% similarity, while *D. stramonium* was somewhat more similar to *D. ferox* × f. *stramonium* (67%) than to *D. ferox* × f. *tatula* (60%). The two species were similar at a level of 46% while *D. ferox* × f. *stramonium* and *D. ferox* × f. *tatula* share 86% similarity. In accordance, Dymshakova *et al*. [27], using genetic distances estimated by AFLPs, showed that F_1_ hybrids were intermediate between the parentals *Saxifraga sibirica* and *S. cernua*. It is clear from the results that the putative hybrids were highly similar to each other, meaning that share common ancestry, and a high percentage of their genetic makeup is equally similar to both *D. stramonium* and *D. ferox* at a level higher than 60%, and hence those were possibly their progenitors. This evidence supports the hypothesis that the two intermediate accessions may be inter-specific hybrids between *D. ferox* and *D. stramonium*.

## Conclusions

The natural hybridization of annual *Datura* species confers putative implications in biological and agronomic terms. In extreme, it could be the first step for speciation but more possibly, it could change local population composition, which in turn could affect *Datura* weed competitiveness and its susceptibility to chemical or mechanical control. Finally, this hybridization could raise alkaloid content leading to commercial interest of its extraction.

## Methods

### Site description and morphological measurements

During September 2011, mixed swards of *Datura* species were identified at the locales of Eukarpia (40°32′N, 22°60′E, 14 m a.s.l.) and Agios Demetrios (40°53′N, 23°41′E, 66 m a.s.l.) in Serres region, northern Greece. The predominant *D. ferox* was coexisting with the typical green-stemmed, white-flowered *D. stramonium* f. *stramonium* (f. *stramonium*) and the purple-stemmed, violet-flowered *D. stramonium* f. *tatula* (f. *tatula*). The swards emerged postharvest, after erratic summer rains, in winter cereal fields. The preceding crop was cotton, the spring crop where *D. ferox* mainly resides. The soil of the fields in the wider area was derived from alluvial deposits and the climate is Mediterranean, with dry and hot summers.

**Figure 4:**
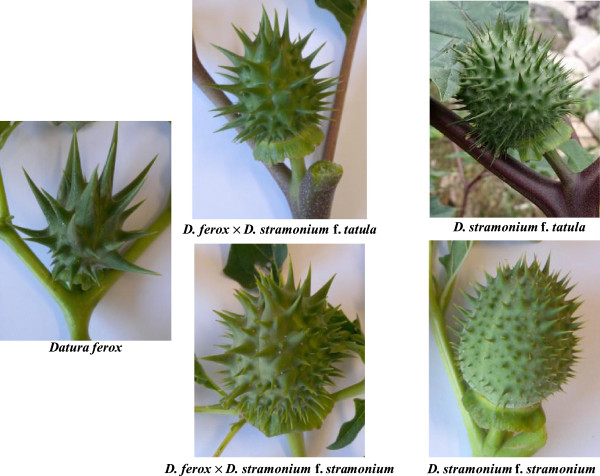
**Upper bearing fork and capsule of the putative parents and their hybrids.**

Determinations were conducted *in situ* at Eukarpia site, where *Datura* population cohort was more homogenous. Six specimens, typical of each variant (Figure 4), were selected for measurements, which were conducted on the upper full expanded leaves (length, width, and length/width ratio), flowers (corolla and calyx length) and capsules (length, width, number of spines, short spine length, medium spine length, and long spine length). Lengths were measured using a HOREX electronic digital caliper (Helios-Preisser, Gammertingen, Germany). Stem color of the specimens was also recorded.

### Plant material sampling and molecular determinations

The plant samples included *Datura ferox* (F), *D. stramonium* forms (S), and their putative inter-specific hybrids (*D. ferox* × f. *tatula*: A and *D. ferox* × f. *stramonium*: B).

Upper forks of each specimen were selected, sealed in a plastic bag, put in a portable refrigerator and transferred to the laboratory for DNA extraction, which was performed from 100 mg ground leaf tissue using the cetyl trimethylammonium bromide (CTAB) method according to the protocol outlined in the NucleoSpin®Plant II kit (Macherey Nagel GmbH & Co. KG, Düren, Germany).

### Oligonucleotide primers and PCR conditions for fingerprinting and analysis of the cpDNA trnL intron

A KAPA Taq PCR kit was used to perform the PCR reactions (Kapa Biosystems Inc., Wilmington, USA). The PCR mixture in a total volume of 25 μl contained 1× PCR buffer A (containing MgCl_2_), separate MgCl_2_ solution, so that total Mg concentration was fixed at 3.0 mM, 0.2 mM of each dNTP (New England Biolabs, Ipswich, USA), 2.4 μM of each primer (forward and reverse) and 1.2 U KAPA Taq polymerase. The amount of genomic DNA added in the PCR mixture was about 250 ng. PCR amplification was carried out in a Veriti 96Well Thermal Cycler (Applied Biosystems, Foster City, USA) using the following profile: a first step of 3 min at 94°C; a second step of 10 cycles of 30 sec at 94°C, 45 sec at 56°C (touchdown: -1°C per cycle), 3 min at 72°C; a third step of 30 cycles of 30 sec at 94°C, 45 sec at 47°C, 3 min at 72°C; and a step of a final extension for 11 min at 72°C. For fingerprinting the different genotypes, 12 universal rice primers (URP) were used [28]. URPs are 20-mer primers that were designed for fingerprinting rice genomes, based on the sequence of a rice-specific CACTA-like transposon element. The sequence of this repeated element (named pKRD) was not found in other plant, animal or fungal genomes [30]. Despite that, sequences homologous to URP over a continuous range of 15 bases were widely observed on diverse genomes including *Arabidopsis*, human and bacteria [28]. Thus, URP primers were used for fingerprinting other organisms [28], and were proved a useful tool for genetic characterization and grouping of most eukaryotic or prokaryotic genomes, especially at inter- and intraspecific levels [31, 32]. The URP are listed in Table 5. DNA fragments were detected by staining with ethidium bromide on a 3% MetaPhor™ agarose (Cambrex Bio Science, Copenhagen, Denmark) gel in TBE buffer.

**Table 5:** **Characteristics of 12 URP and the P1 and P2 primers used for amplifying trnL intron**

No	Primers	Sequences (5′-3′)	GC% content	Tm (°C)
1	URP1F	ATCCAAGGTCCGAGACAACC	55	65
2	URP2F	GTGTGCGATCAGTTGCTGGG	60	67
3	URP2R	CCCAGCAACTGATCGCACAC	60	65
4	URP4R	AGGACTCGATAACAGGCTCC	55	66
5	URP6R	GGCAAGCTGGTGGGAGGTAC	65	65
6	URP9F	ATGTGTGCGATCAGTTGCTG	50	67
7	URP13R	TACATCGCAAGTGACACAGG	50	68
8	URP17R	AATGTGGGCAAGCTGGTGGT	55	74
9	URP25F	GATGTGTTCTTGGAGCCTGT	50	65
10	URP30F	GGACAAGAAGAGGATGTGGA	50	65
11	URP32F	TACACGTCTCGATCTACAGG	50	65
12	URP38F	AAGAGGCATTCTACCACCAC	50	65
13	P1	CGAAATCGGTAGACGCTACG	55	63
14	P2	GGGGATAGAGGGACTTGAAC	55	62

For amplification of the cpDNA trnL intron, the PCR primes P1 and P2 were used (Table 5). PCR setup and instruments were as above. Amplification profile was: one cycle of 2 min at 94°C; 10 cycles of 30 sec at 94°C, 30 sec at 58°C (touchdown: -0.5°C per cycle), 50 sec at 72°C; 30 cycles of 30 sec at 94°C, 30 sec at 53°C, 50 sec at 72°C; one cycle of a final extension for 10 min at 72°C. DNA fragments were detected by staining with ethidium bromide on a 3% MetaPhor™ agarose (Cambrex Bio Science, Copenhagen, Denmark) gel in TBE buffer. The bands of the trnL intron were excised from the gel, DNA was recovered using a modified freeze-squeeze method [33] and sent for direct sequencing with the primers used in PCR.

### Statistical analysis of morphological characteristics

With the exception of corolla and calyx lengths in *D. ferox* × f. *tatula* for which measurements were not taken due to the lack of appropriate samples, morphological trait means and standard errors were computed for summarizing the distributions of the corresponding variables. On the basis of morphological data (except stem colour), the five variants were compared with the Analysis of Variance (ANOVA) method. The Duncan’s multiple range test was used for means’ comparisons.

Principal Components Analysis (PCA) with varimax rotation was applied on the correlation matrix between the morphological variables (except corolla and calyx lengths) in order to study the groupings, similarities, and differences between the individuals of the five variants. Significant components were determined by the parallel analysis (PA) method [34]. Since the stem colour was a nominal categorical variable, with three categories (purple, green, and grey-green), two dummy variables with binary coding (0, 1) were entered in the PCA; one variable for the purple colour and one for the green. A third dummy variable for the grey-green colour is redundant since it would be dependent on and negatively correlated with the other two dummy variables. Generally, the number of dummy-coded variables needed is one less than the number of modalities of the corresponding categorical variable. Since PCA is not a modelling but only a descriptive variance summarizing method, the use of binary along with scale variables is legitimate [35]. The significance level for all hypotheses testing procedures was preset at *p* < 0.05. IBM SPSS package v. 20 (IBM Corp., New York, USA) was used for the analyses. Parallel analysis was conducted with the RanEigen v. 2.0 software [36].

### Analysis of molecular data

Gel photographs were scored using the gel image analysis software GelAnalyzer 2010a (http://www.gelanalyzer.com/). The bands were binary coded with 1 or 0 for their presence or absence in each genotype, respectively, and the coded data were subjected to statistical analysis. Estimates of similarity among all genotypes were calculated from the Jaccard’s similarity coefficient using the IBM SPSS package v. 20 (IBM Corp., New York, USA). Hierarchical cluster analysis based on the Jaccard similarity matrix with the unweighted pair group method based on arithmetic averages (UPGMA) [37] was conducted using the software MEGA5 [38].

## Abbreviations

AFLP: Amplified fragment length polymorphism
ANOVA: Analysis of variance
CaL: Calyx length
CL: Capsule length
CoL: Corolla length
CTAB: Cetyl trimethylammonium bromide
CW: Capsule width
LL: Leaf length
LSL: Long spike length
LW: Leaf width
MSL: Medium spike length
PA: Parallel analysis
PC: Principal component
PCA: Principal components analysis
PCR: Polymerase chain reaction
RAPD: Random amplified polymorphic DNA
SC: Stem color
SSL: Short spike length
SSR: Simple sequence repeat
UPGMA: Unweighted pair group method based on arithmetic averages.
