# Correction: in the wild hybridization of annual datura species as unveiled by morphological and molecular comparisons

**DOI:** 10.1186/2241-5793-21-18

**Published:** 2014-11-07

**Authors:** Ioannis T Tsialtas, Efstathia Patelou, Nikolaos S Kaloumenos, Photini V Mylona, Alexios Polidoros, Georgios Menexes, Ilias G Eleftherohorinos

**Affiliations:** Faculty of Agriculture, Laboratory of Agronomy, Aristotle University of Thessaloniki, 541 24 Thessaloniki, Greece; Faculty of Agriculture, Laboratory of Genetics and Plant Breeding, Aristotle University of Thessaloniki, 541 24 Thessaloniki, Greece; Biological Sciences, Syngenta, Jealott’s Hill International Research Centre, Bracknell, Berkshire, RG42 6EY UK; ELGO-“Demetra”, Agricultural Research Center of Northern Greece, 570 01 Thermi, Greece

## Correction

After the publication of this work [1], it was brought to the author’s attention that in the sentence “Genus *Datura*, family Solanaceae, consists of nine (annual and tree) species, originating from the New and Old World [1]”, “originating from” should be replaced with “present in”. We regret any inconvenience that this inaccuracy may have caused.
